# Trauma, mind and brain: the impact of war on mental health

**DOI:** 10.1192/j.eurpsy.2023.27

**Published:** 2023-07-19

**Authors:** A. Maercker

**Affiliations:** Dept. of Psychology, University of Zurich, Zurich, Switzerland

## Abstract

**Abstract:**

The PTSD diagnosis was in 1980 largely facilitated by consequences of the Vietnam War. Since then, there have been hundreds of other war-related conflicts in the world. My presentation will distinguish between war-related trauma effects on military personnel (where most research has been done) and on civilians, distinguishing effects on children/adolescents, adults and older people. I will answer the question, how do war-related trauma sequelae differ from other man-made or accidental traumas? Further, I will address the issue of whether “moral injury” research in military personnel after wartime operations is also relevant to similar phenomena in civilian populations traumatised by war? Which of the brain-related research approaches (localisation, network connectivity, altered RDoC functions) are particularly relevant in this context? Finally, emerging research priorities related to the current war of invasion against Ukraine will be addressed.

**Disclosure of Interest:**

None Declared

